# Canine coronavirus infection is intensified by 2,3,7,8-tetrachlorodibenzo-*p-*dioxin

**DOI:** 10.1007/s00204-025-03981-w

**Published:** 2025-02-22

**Authors:** Luca Del Sorbo, Claudia Cerracchio, Francesco Serra, Silvia Canzanella, Rosa Giugliano, Sara Lambiase, Nicolás Pizarro Aránguiz, Mauro Esposito, Maria Grazia Amoroso, Giovanna Fusco, Filomena Fiorito

**Affiliations:** 1 Department of Veterinary Medicine and Animal Production, University of Naples Federico II, 80137 Naples, Italy; 2 Istituto Zooprofilattico del Mezzogiorno, Portici, 80055 Naples, Italy; 3 Instituto de Investigaciones Agropecuarias, INIA Remehue. Ruta 5 Km 8, 7500502 Osorno, Chile

**Keywords:** CCoV, TCDD, Viral N protein, Virus yield, AHR signaling

## Abstract

In humans as well as in animals, the toxic contaminant 2,3,7,8-tetrachlorodibenzo-*p-*dioxin (TCDD) stimulates immunosuppression and increases responsiveness to infectious diseases. The relationship between environmental contaminants and different infectious diseases, including COVID-19, has been described. Nevertheless, reports about the potential impact of TCDD on coronaviruses (CoVs) are limited. In this study, the impact of TCDD (0–100 pg/mL) was assessed during infection in vitro with canine coronavirus (CCoV-II), the alphaCoV causing moderate enteric disease in dogs, although genetic alterations may surprisingly generate new dangerous strains. For instance, outbreaks of lethal infections in dogs were related to highly virulent CCoV strains, and cases of pneumonia and malaise in humans were associated with new canine–feline recombinant strains of CCoV, underlining the cross-species spread capability of CoVs. Herein, during CCoV infection, TCDD induced a substantial growth in virus yield and in the expression of viral nucleocapsid protein in infected groups. Infected cells exhibited alterations in cell morphology, extensively enhanced by TCDD. Moreover, in infection, TCDD modulated the protein levels of aryl hydrocarbon receptor (AHR), a signaling responsive to both environmental contaminant and CoVs infections. Overall, our findings showed that TCDD, playing a role in AHR signaling, may worsen CCoV infection.

## Introduction

CoVs can cause infection in several hosts including mammals and birds (Kenney et al. 2021; Pratelli et al. 2022). Mutation and recombination are mechanisms used by CoVs to generate new and more dangerous strains, allowing them to overcome the natural barriers promoting cross-species transmission (Kenney et al. 2021; Pratelli et al. 2021, 2022). CCoV-II, an alphaCoV belongs to *Coronaviridae* family, is generally responsible for enteritis in dogs with high morbidity and low mortality, a disease resolvable without treatment (Pratelli et al. 2021, 2022). However, in 2005, a particularly dangerous CCoV-IIa strain, namely as pantropic, was recognized as the agent responsible for multi-systemic infections in dead puppies (Buonavoglia et al. 2006), and finally, new recombinant strains of CCoV-II, due to a partial involvement of the 5’end S-gene which resulted exchanged with transmissible gastroenteritis virus (TGEV), were also detected in dead pups (Decaro et al. 2007, 2009). Recently, a noteworthy strain of CCoV was detected from samples of nasal secretions of Malaysian children hospitalized for pneumonia. Genetic analysis of the virus, namely CCoV-HuPn-2018, has been demonstrated the presence of canine–feline-like recombination, including a single gene deletion in the nucleoprotein (NP), close to the one identified in humans CoVs like severe acute respiratory syndrome CoV type 1 (SARS-CoV-1) and SARS-CoV-2, indicating that CCoV-HuPn-2018 has jumped from animals to humans (Vlasova et al. 2022a). Furthermore, HuCCoV_Z19Haiti, a novel CoV, was identified from the urine samples of a sick healthcare component after a mission to Haiti. Genetic investigation displayed a notable (99.4%) affinity of HuCCoV_Z19Haiti compared to CCoV-HuPn-2018 (Lednicky et al. 2021). Surprisingly, human alphaCoVs including canine–feline-porcine recombination were also separately noticed in Thailand (Theamboonlers et al. 2007) and USA (Silva et al. 2014), suggesting that these CoVs occurred in distinct countries (Vlasova et al. 2022b). In CoVs–host interaction, it has recently established a role for the transcription factor aryl hydrocarbon receptor (AHR), that is regulated by different substances, along with environmental contaminants and microbial metabolites (Barreira-Silva et al. 2024; Hu et al. 2023). After binding, the complex AHR molecule translocases into the nucleus, where it modulates genetic expression of the Cytochrome P450 family enzymes like CYP1A1, provoking changes in cytokines levels and immunity regulation (Barreira-Silva et al. 2024; Hu et al. 2023). In infection, AHR can modify natural immunological reactions to various pathogens (Barreira-Silva et al. 2024; Hu et al. 2023), including CoVs like Middle East respiratory syndrome coronavirus (MERS-CoV), mouse hepatitis virus (MHV), human coronavirus (HCoV) 229E, HCoV-OC43, SARS-CoV-1, SARS-CoV-2, and CCoV-II (Grunewald et al. 2020; Giovannoni et al. 2021; Cerracchio et al. 2022; Shi et al. 2023; Yousefi et al. 2023; Healey et al. 2024). Precisely, the inhibition of MCoV infection, induced by a selective AHR inhibitor (CH223191), reduces the expression of cytokines interleukin (IL)-1β and (IL)-10, as well as upregulates the levels of tumor necrosis factor-alpha, while TCDD reverses this modulation (Grunewald et al. 2020).

Among environmental contaminants, polychlorinated dibenzo-*p-*dioxins (PCDDs) and polychlorinated dibenzofurans (PCDFs) are a family of 210 aromatic organochlorine congeners, and TCDD is the prototype for the family. Resistance to metabolism, photo-degradation, and microbial processes are characteristics which contribute to determine the remarkable persistence of PCDD/PCDFs, which bioaccumulate in environment provoking toxic effects both in humans and animals (Fiorito et al. 2017b; Eskenazi et al. 2018; Bock 2018; European Food Safety Authority [EFSA] 2018; Mandal 2023; Kanan et al. 2025). PCDD/PCDFs can be released by natural emissions such as volcanic eruptions, and they are a byproduct of some manufacturing processes, including pesticides, herbicides, insecticides, fungicides, wood preservatives, and chlorine bleaching of paper pulp. However, the main source of emissions of PCDD/PCDFs is due to incineration plants (municipal, medical, and hazardous wastes) (Fiorito et al. 2017b; Eskenazi et al. 2018; Bock 2018; EFSA 2018; Domingo et al. 2002, 2020a; 2024; Marquès and Domingo 2019; Nadal et al. 2019, 2020; García et al. 2021; Mandal 2023; Kanan et al. 2025). Indeed, epidemiological surveys highlight that living in proximity to municipal solid waste incinerators (MSWI) or hazardous waste incinerators (HWIs) may induce several serious health effects including cancers. Thus, to ensure the safety of humans living nearby incinerators as well as to guarantee that the MSWIs and HWIs work within great environmental standards, monitoring studies are indispensable to analyze emissions, not only of PCDDs and PCDFs, but also of heavy metals and other chemicals, unknown or not usually examined for the management of hazardous waste (Marquès and Domingo 2019; Nadal et al. 2019, 2020; García et al. 2021; Domingo et al. 2002, 2020a; 2024). Moreover, emissions of PCDD/PCDFs are also caused by illegal burning of industrial and urban waste, like in the Land of Fires (Campania region, Italy) (EFSA 2018; Ssebugere et al. 2019; Alberti 2022; Esposito et al. 2010; Lambiase et al. 2022, 2024), also known as the *Terra dei Fuochi*, *where some 2.9 million people live.* Particularly, *increased rates of cancer and pollution of groundwater had been recorded in the area*, and during the preparation of this manuscript, the European Court of Human Rights held, unanimously, that there had been *a violation of Article 2 (right to life) of the European Convention on Human Rights* as* “prolonged inaction by Italian State on widespread dumping put Terra dei Fuochi residents’ lives at risk*” (Judgment concerning Italy—ECHR—ECHR / CEDH).

PCDD/PCDFs may reach humans by inhalation (10%) and for non-occupationally exposed humans by food ingestion (90%) of animal origin, including milk or milk products, fish, eggs, and some vegetables (González et al. 2020; EFSA 2018). Among PCDD/PCDFs, due to their persistence, 17 congeners are considered relevant in animals and humans, and for which the World Health Organization in 2005 has assigned toxicity equivalency factors (EFSA 2018). The compounds of the PCDD/PCDFs family are liposoluble substances that, by biomagnification in the food chain, accumulate in adipose organs and tissues. They are structurally related and provoke toxicity through a mechanism of action involving AHR, resulting in a large range of dangerous actions in humans as well as in animals (Fiorito et al. 2017b; Eskenazi et al. 2018; Bock 2018; EFSA 2018; Coelho et al. 2022; Mandal 2023; Kanan et al. 2025).

Due to carcinogenic studies, TCDD, the most toxic congener in the family PCDD/PCDFs was categorized as human cancer promoter into Group 1 by the World Health Organization's International Agency for Research on Cancer (IARC) (Agents Classified by the IARC Monographs, Volumes 1–137—IARC Monographs on the Identification of Carcinogenic Hazards to Humans). Thus, TCDD is the most studied congener in the family PCDD/PCDFs, and numerous reports have described effects like cancer (breast, prostate, and thyroid). TCDD also induce endocrine disrupting effects, skin diseases, liver toxicity, cardiometabolic diseases, neurodevelopmental impairment, toxicity on reproductive organs, and teratogenesis. In addition, TCDD may cause thymic atrophy and immunosuppression, resulting in increased susceptibility to infectious agents (Singh et al. 2022; Sabuz Vidal et al. 2021; Wang et al. 2023; Bock 2018; Fiorito et al. 2017a). Thus, TCDD can induce a worsened host response to different viruses. For example, following infection with MHV (Healey et al. 2024), and various subtypes of influenza viruses (House et al. 1990; Burleson et al. 1996; Bohn et al. 2005), the exposure to TCDD enhanced morbidity or mortality of infected mice. TCDD also induces an enhancement of virus replication in different cell lines, during infection with MHV in murine bone-marrow-derived macrophages (BMDMs) (Grunewald et al. 2020), human immunodeficiency virus-1 (HIV-1) (MT-4 and U1cells) (Pokrovsky et al. 1991; Gollapudi et al. 1996), cytomegalovirus (CMV) (MRC-5 cells) (Murayama et al. 2002), as well as in bovine herpesvirus 1 (BoHV-1) (MDBK) (Fiorito et al. 2008a, b, 2010, 2017a).

In this scenario, among the congeners of PCDD/PCDFs, TCDD was selected with the aim of studying the influence of a persistent environmental contaminant, like TCDD, in host responses to CoVs infections. Up to now, only very few reports have evaluated the effects of TCDD in CoVs infections (Grunewald et al. 2020; Healey et al. 2024). Hence, we evaluated how TCDD has effect over CCoV infection in vitro, exploring AHR signaling.

## Materials and methods

### Cell cultures and virus infection

A72 cells were cultured in Dulbecco’s modified Eagle's minimal essential medium (DMEM) and incubated, as described (De Martino et al. 2010; Marfè et al. 2011). CCoV type II (strain S/378, GenBank accession number KC175341) was utilized.

Virus stocks’ growth as well as virus titration were performed on A72 cells (De Martino et al. 2010).

TCDD in DMSO (10^–4^ M) (Wellington Laboratories Inc., Ontario, Canada). A stock solution of TCDD (10,000 pg/mL) was prepared by dissolving it in DMSO (Sigma-Aldrich, St. Louis, MO, USA). Subsequently, working solutions of 0.01, 1, and 100 pg/mL in DMEM were obtained and added to culture cells, according to our previous studies (Fiorito et al. 2008a, b, 2010, 2011, 2017a). DMSO in DMEM (0.1% v/v) was used as vehicle control.

About the presence of PCDD/Fs in feed and food, EFSA, in the Scientific Opinion on the risks for animal and human health, established the tolerable weekly intake (TWI) of 2 pg TEQ kg^−1^ body weight week^−1^ (EFSA 2018). Thus, in this study, we used concentrations of TCDD lower and upper TWI. Moreover, these concentrations were previously reported in in vitro studies (Fiorito et al. 2008a, b, 2010, 2017a).

A72 cells were treated with TCDD (0, 0.01, 1, and 100 pg/mL) or DMSO (TCDD 0 pg/mL). In addition, A72 cells were infected with CCoV, at multiplicity of infection (MOI) of 0.1, 1, or 10, and treated with TCDD (0.01, 1, and 100 pg/mL) or DMSO (TCDD 0 pg/mL), to have eight groups: DMSO treated (TCDD 0 pg/mL) infected and uninfected cells, TCDD (0.01 pg/mL) treated infected and uninfected cells, TCDD (1 pg/mL) treated infected and uninfected cells, and TCDD (100 pg/mL) treated infected and uninfected cells. After adsorption at 37 °C for 1 h, cells were incubated and handled at 0, 1, 3, 6, 12, 24, 48, and 72 h post-infection (p.i.). CCoV was in the culture medium during the experiment.

### Cell viability assay

Cell Proliferation Kit I 3-(4,5-dimethyl-2-thiazolyl)-2,5-diphenyl-2H-tetrazolium bromide (MTT) (Roche, Basel Switzerland) assay was used to assess cell viability (Fiorito et al. 2008b, 2022). In brief, cells were exposed to TCDD (0, 0.01, 1, and 100 pg/mL), and incubated. After 24 h of TCDD exposure, MTT assay was performed, measuring the absorbance at 540 nm (A_540_). Results were shown as the mean ± S.D. of four independent experiments in duplicate.

### Investigation in cell morphology

To investigate the influence of TCDD on A72 cell morphology, Giemsa staining and acridine orange/propidium iodide (AO/PI) were achieved (Cerracchio et al. 2023, 2024). Cells were infected with CCoV, at MOI of 1, in the presence of TCDD (0, 0.01, 1, and 100 pg/mL) and incubated at 37 °C. At 24 h p.i., Giemsa staining was carried out, and light microscopy examination was performed under ZOE Cell Imager (Bio-Rad Laboratories, Hercules, CA, USA). Morphological features of cell death were recognized using the specific instructions (Leite et al. 1999; Banfalvi 2017).

### Immunofluorescence staining

Cells were infected with CCoV at MOI 0.1 and 10 and exposed to TCDD (0, 0.01, 1, and 100 pg/mL). After 24 h of infection, immunofluorescence staining (IF) was assessed as previously demonstrated (Altamura et al. 2018; Fiorito et al. 2022). The following antibodies were used: anti-AhR (Sigma-Aldrich, St. Louis, MO, USA) (1:250), anti-NP monoclonal mouse, MAB 938 (The Native Antigen Company, Kidlington, UK), monoclonal mouse anti-CYP1A1 (A-9) (sc-393979, Santa Cruz Biotechnology, Inc., Dallas, TX, USA) (1:250); Texas Red goat anti-rabbit (Thermo Fisher Scientific, Waltham, MA, USA) (1:500), Alexa Fluor 488 goat anti-mouse (Thermo Fisher Scientific, Waltham, MA, USA) (1:1000). Nuclear counter-staining was assessed by DAPI (1:1000). Microscopy and photography were evaluated by ZOE Fluorescent Cell Imager (Bio-Rad Laboratories, Hercules, CA, USA). Fluorescence signals from microscopy-generated images were analyzed by ImageJ (National Institutes of Health, Bethesda, MD, USA) software.

### Cells viral infection

Cells were infected with CCoV at MOI 10, in the presence of different doses (0, 0.01, 1, and 100 pg/mL) of TCDD, incubated at 37 °C and cell supernatant analyzed for CCoV quantification at 0, 1, 3, 6, 12, 24, 48, and 72 h p.i. by real-time PCR. Furthermore, viral cytopathic effect (CPE) was assessed at 24 h of infection, after washing, fixing, and staining cells with crystal violet (Murphy et al. 2018; De Martino et al. 2010).

### Viral nucleic acids’ extraction

Nucleic acids’ extraction on cell supernatant (200 µL) was performed by QIAsymphony automated extraction system (Qiagen GmbH, Hilden, Germany) with the DSP Virus/Pathogen Mini kit (Qiagen GmbH, Hilden, Germany) following the manufacturer’s instructions. Then, the elution of nucleic acids in elution buffer (80 µL) and the analysis by Real-Time PCR were assessed. DMEM was the negative process control.

### Real-time PCR for quantification of virus

Quantitative real-time PCR was carried out to quantify CCoV in all the samples. Reaction was assessed on a Quant Studio 5 Real-Time PCR thermal cycler (Thermo Fisher Scientific, Waltham, MA, USA) in a total volume of 25 µL containing: nucleic acids extract (5 µL), AGPATH reaction kit (12.5 µL), reverse transcriptase enzyme (1 µL) (Thermo Fisher Scientific, Waltham, MA, USA), primer forward CCoV-For (5'-TTGATCGTTTTTATAACGGTTCTACAA-3') (1 µL at 10 µM), primer reverse CCoV-Rev (5'-AATGGGCCATAATAGCCACATAAT-3') (1 µL at 10 µM), and probe CCoV-P (FAM-5'-ACCTCAATTTAGCTGGTTCGTGTATGGCATT -3'-TAMRA) (1 µL at 6 µM). The thermal profile included a step of reverse transcription (30 min at 42 °C), initial denaturation (15 min at 95 °C), and 40 cycles including a denaturation (15 s at 95 °C) and an annealing/extension (60 s at 60 °C) (Decaro et al. 2010). A standard curve, developed by amplifying serial dilutions of the quantified extracted virus (from 3.5 × 10^9^ to 3.5 × 10^5^ TCID_50_/mL) and plotting Log (TCID_50_/ml) versus the threshold cycle (Ct), was used to quantify the virus. The efficiency of amplification (E) was determined as previously reported (Amoroso et al. 2011). To analyze the results, samples with Ct < 40 were considered positive, expressing the quantification as TCID_50_/mL.

### Statistical analysis

All the results and graphs show means and standard deviations (SD). GraphPad InStat Version 3.00 for Windows 95 (GraphPad Software, San Diego, CA) was used to determine one-way ANOVA with Tukey’s post-test. *p* value < 0.05 was statistically significant. The quantity of biological replicates for each experiment is described in each figure.

## Results

### TCDD does not affect A72 cell viability

**Fig. 1:**
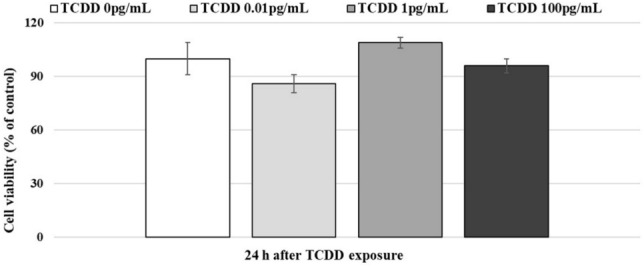
Dose–response curve of A72 cells treated with TCDD (0, 0.01, 1 and 100 pg/mL). A72 cells were exposed to different concentrations of TCDD, and after 24 h of exposure, cell viability was performed by MTT test. *p* > 0.05. Results show the mean ± S.D. of four independent experiments in duplicate

To evaluate cell viability after exposure to TCDD at different concentrations (0, 0.01 pg/mL, 1 pg/mL, and 100 pg/mL), MTT test was performed. No significant (*p* > 0.05) changes were found after exposing A72 to TCDD for 24 h (Fig. 1).

These findings showed that TCDD (0.01, 1 and 100 pg/mL) exposure does not provoke significant differences in A72 cell viability.

### TCDD increases the typical features of morphological cell death induced by CCoV infection in A72 cells

**Fig. 2:**
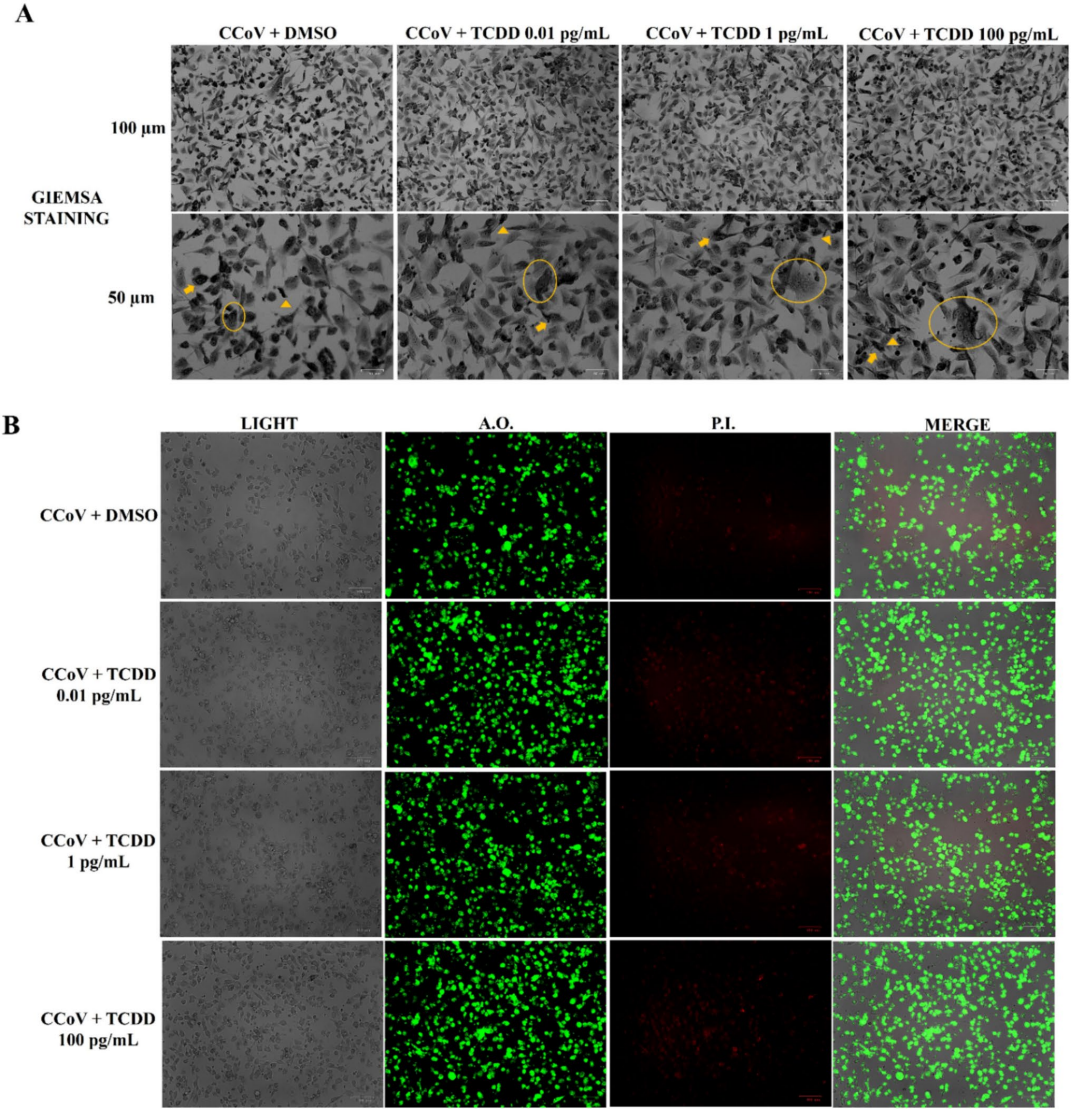
Cell morphology of A72 cells infected with CCoV and treated with DMSO or TCDD (0.01, 1, and 100 pg/mL). A72 infected with CCoV were treated with TCDD for 24 h, stained by Giemsa or AO/PI, and analyzed by light microscopy. **a** In Giemsa staining panels, pyknosis, as well as condensation of chromatin (arrow), cell shrinkage (arrowhead), and cell clusters (circle) were observed. **b** In AO/PI pictures, fluorescent cells (PI) were identified. Scale bar 50 µm and 100 µm. The data show the results of one experiment representative of three independent experiments

The influence of TCDD during CCoV infection on cell morphology was assessed by Giemsa and AO/PI staining. After 24 of CCoV infection in A72, morphological signs of cell death like cellular shrinkage (Fig. 2A, arrowhead), pyknosis (Fig. 2A, arrow), and cell cluster (Fig. 2A, circle) were remarkably improved by TCDD (Fig. 2A). These effects were confirmed using AO/PI. Viable and dead cells can be easily detected by fluorescence microscopy, using these fluorochromes. Indeed, due to membrane permeability, AO binds nucleic acids, triggering green fluorescence. PI, that does not cross the intact cell membrane, crosses the membrane of dead and dying cells, where intercalating nucleic acids arises bright red fluorescent complexes (Cerracchio et al. 2024). A dose-dependent increase in PI fluorescent cells was detected in infected cells exposed to TCDD compared to unexposed-infected groups (Fig. 2B).

Taken together, these results showed that TCDD provoked an increase in the typical features of morphological cell death (necrosis and apoptosis) typically induced by CCoV infection in A72 cells.

### TCDD enhances virus yield during CCoV infection in A72 cells

**Fig. 3:**
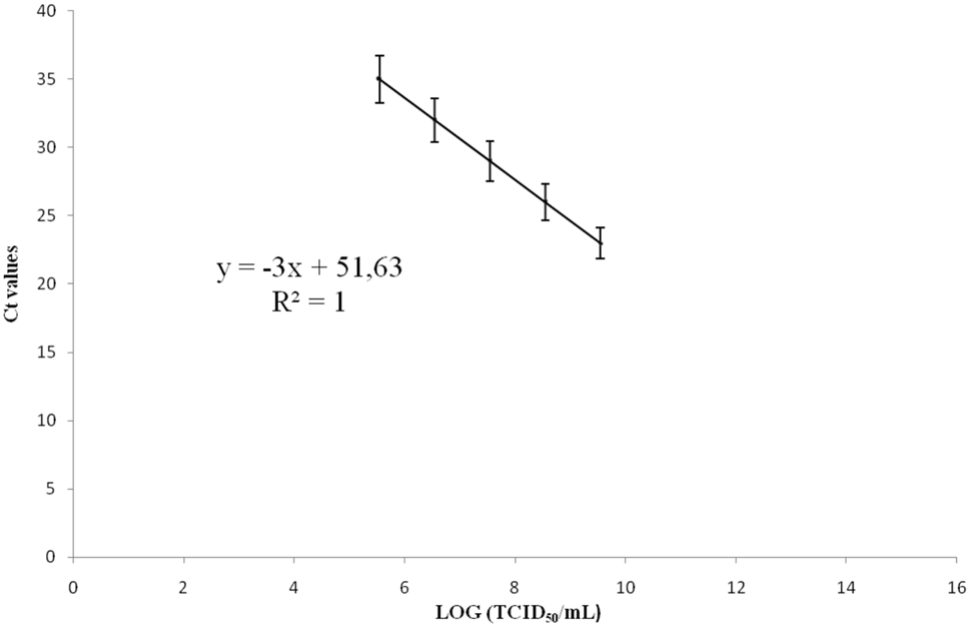
Standard curve found by Real-Time PCR obtained through the analysis of serial dilutions (from 3.5 × 10^9^ to 3.5 × 10^5^ TCID_50_/mL) of CCoV viral stock drawing the Ct vs. Log (TCID_50_/mL)

Viral infection trials were carried out in duplicate and repeated twice. Viral load (TCID_50_/mL) was calculated at different time post-infection (0, 1, 3, 6, 12, 24, 48, and 72 h) considering the standard curve equation depicted in Fig. 3.

**Fig. 4:**
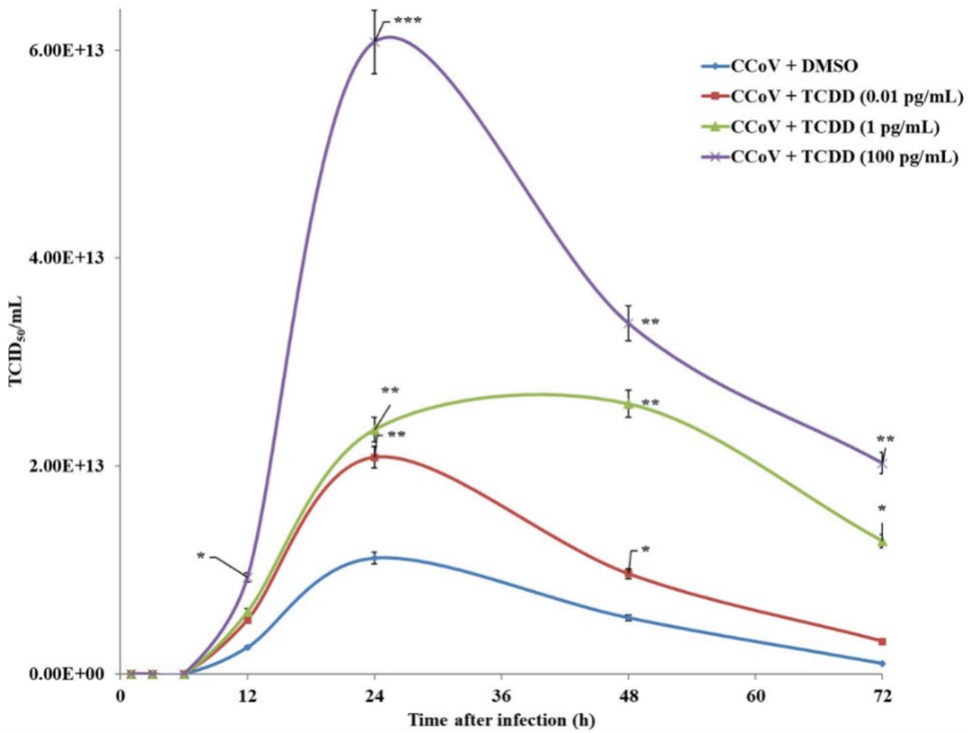
Virus titer of A72 cells infected with CCoV and treated with DMSO or TCDD (0.01, 1, and 100 pg/mL). Cells were infected with CCoV in the presence of DMSO or different doses of TCDD. At 0, 1, 3, 6, 12, 24, 48, and 72 h, virus titer was analyzed by real-time PCR. *p < 0.05, **p < 0.01, and ***p < 0.001

In A72 cells, TCDD significantly increased virus yield, showing time- and dose-dependency (Fig. 4). The influence of TCDD on virus yield was already visible, for the highest concentration, at 12 h post-infection, and continued throughout the experiment. The most remarkable effect (*p* < 0.001) was observed in the presence of 100 pg/mL TCDD at 24 h post-infection, as CCoV viral load in TCDD-exposed infected group was 6.08 × 10^13^ TCID_50_/mL with respect to 1.11 × 10^13^ TCID_50_/mL in unexposed-infected cells.

**Fig. 5:**
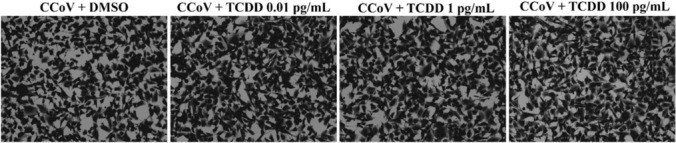
CPE of A72 cells infected with CCoV and treated with DMSO or TCDD (0.01, 1 and 100 pg/mL). A72 cells were infected with CCoV and exposed to DMSO or TCDD (0.01, 1, and 100 pg/mL). After 24 h of infection, CPE was evaluated through crystal violet staining by ZOE Cell Imager. The results of one experiment representative of three independent experiments were displayed

Moreover, after 24 h of infection, the typical CPE induced by CCoV infection in A72 cells was reduced in the presence of TCDD (Fig. 5).

Overall, these results demonstrated that TCDD induces a significant increase in CCoV virus yield.

### TCDD increases the expression of NP in CCoV-infected cells

**Fig. 6:**
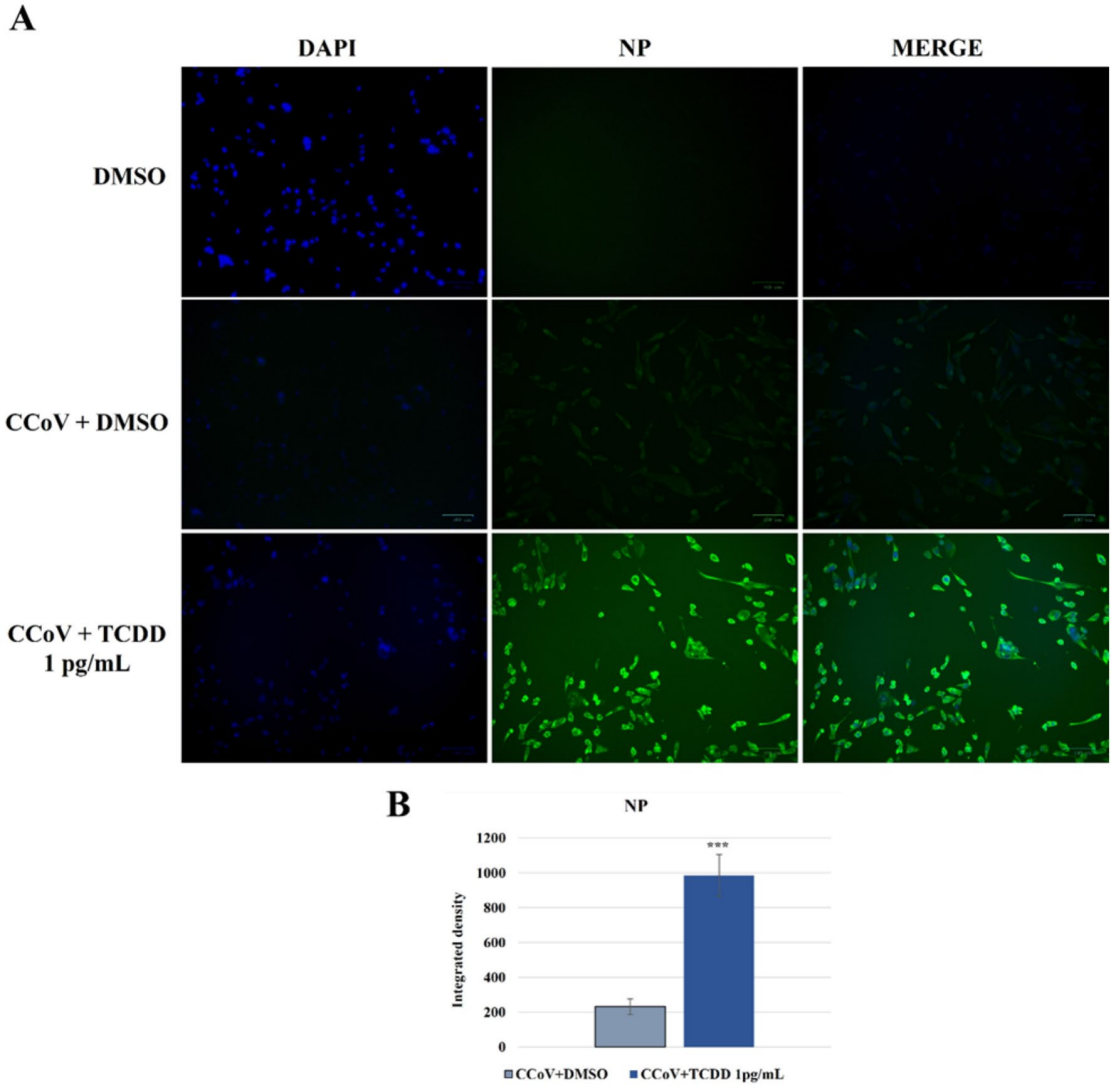
Expression of NP in A72 cells infected with CCoV and treated with DMSO or TCDD (1 pg/mL). Cells were infected with CCoV in the presence of DMSO or TCDD. After 24 h of infection, cells were processed by IF for NP. **A** Fluorescent images of NP. Scale bar 100 µm. **B** Bars are the mean ratio produced from the integrated density (product of the area and mean intensity of fluorescence) of the NP expression assessed by ImageJ. Error bars indicate standard deviation measurement. ***p < 0.001. The data show the results of one experiment representative of three independent experiments

To evaluate nucleocapsid protein (NP) expression, immunofluorescence staining was performed. NP was expressed in nucleus as well as in cytoplasm of infected cells, whose nuclei were weakly labeled with DAPI (Fig. 6). Whereas, in the presence of TCDD, a marked enhance of NP expression was observed in infected cells, whose nuclei were intensely counterstained by DAPI (Fig. 6).

These results showed that during CCoV infection in A72 cells, TCDD provokes a significant increase in the expression of viral NP protein.

### TCDD downregulates the expression of AHR during CCoV infection in A72 cells

**Fig. 7:**
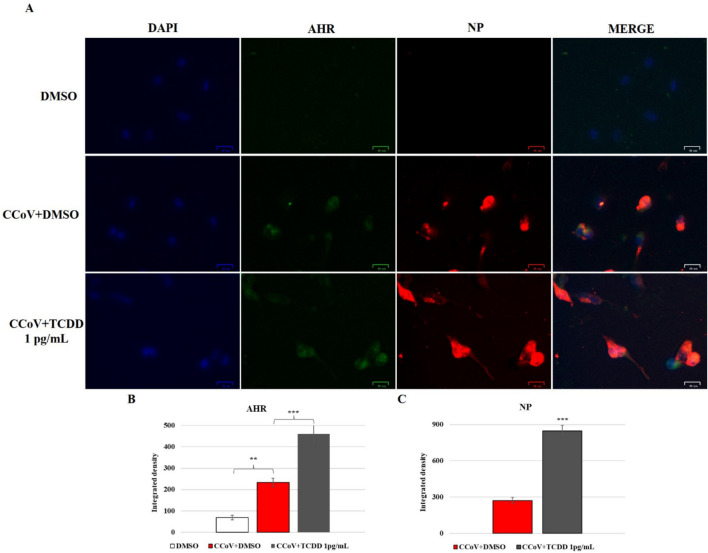
Expression of AHR and NP in A72 cells infected with CCoV and treated with DMSO or TCDD (1 pg/mL). A72 cells were infected with CCoV in the presence of DMSO or TCDD for 24 h and processed by IF for AHR and NP. **A** Fluorescent images of AHR and NP. Scale bar 25 µm. Bars are the mean ratio produced from the integrated density (product of the area and mean intensity of fluorescence) of **B** AHR and **C** NP. The integrated density was determined by ImageJ. Error bars correspond to SD measurement. **p < 0.01 and ***p < 0.001. The figure shows the findings of one experiment representative of three independent experiments

Following CCoV infection, we observed an upregulation in the expression of AHR, which resulted further upregulated by TCDD (Fig. 7). These results were confirmed by integrated density fluorescence analysis (Fig. 7). Whereas the levels of AHR in uninfected groups exposed to TCDD increased (data not shown).

These results showed that CCoV infection in A72 cells activates protein expression of AHR, that resulted further increased by TCDD.

### CCoV infection activates the expression of CYP1A1, which is further increased by TCDD

**Fig. 8:**
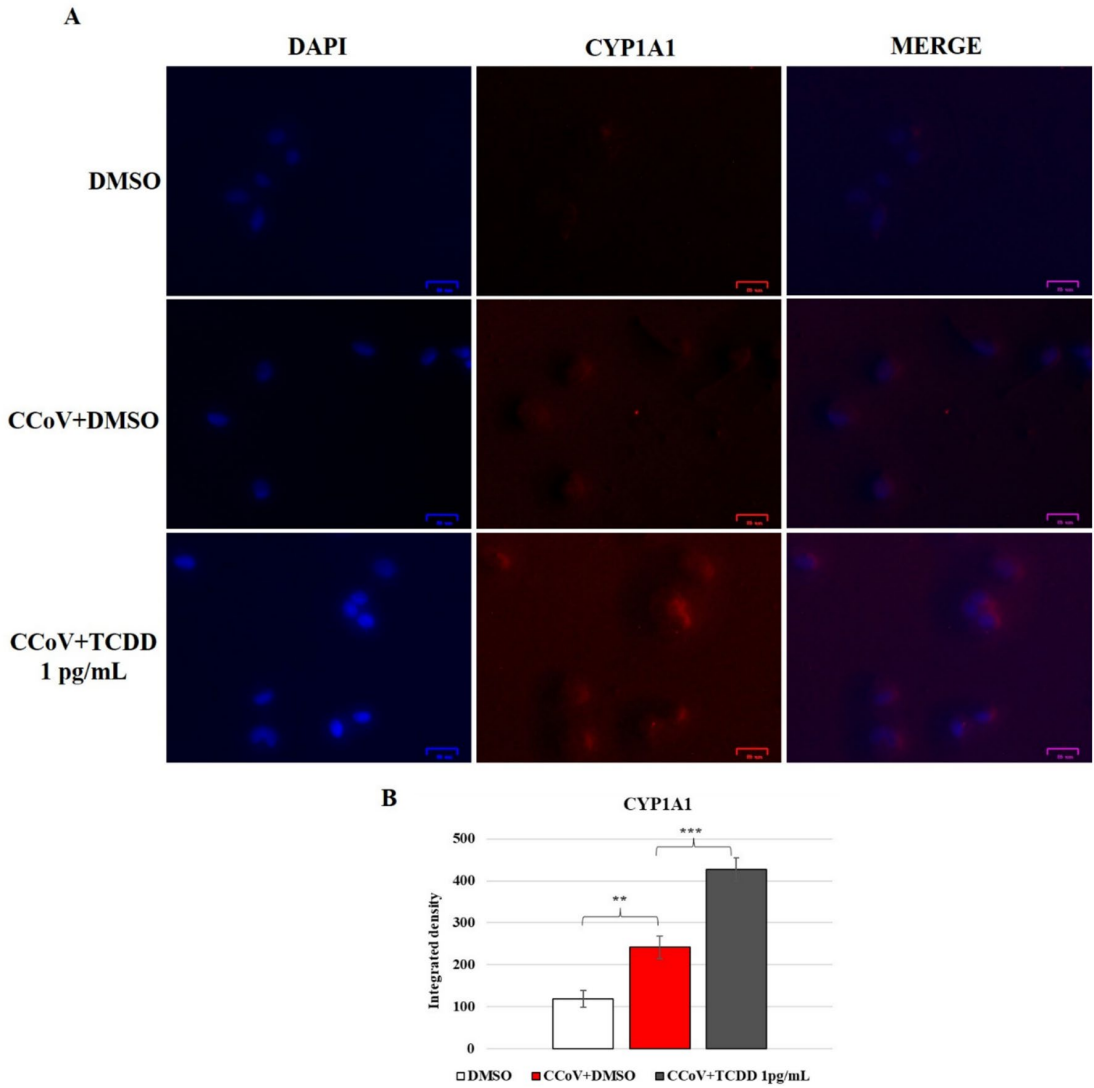
Expression of CYP1A1 in A72 cells infected with CCoV and treated with DMSO or TCDD (1 pg/mL). A72 cells were infected with CCoV in the presence of DMSO or TCDD for 24 h and processed by IF for CYP1A1. **A** Fluorescent images of AHR and CYP1A1. Scale bar 25 µm. **B** Bars are the mean ratio produced from the integrated density (product of the area and mean intensity of fluorescence) of CYP1A1. The integrated density was determined by ImageJ. Error bars represent SD measurement. **p < 0.01 and *p* < *** *p* < 0.001. The data show the results of one experiment representative of three independent experiments

To explore the expression of AHR signaling, the levels of AHR downstream target protein CYP1A1 were evaluated in A72 cells in unexposed-infected groups and in TCDD-exposed cells (Fig. 8). A significant increase in the expression of CYP1A1 was observed in the infected cell groups (Fig. 8), while the TCDD exposure led to a marked down-regulation of CYP1A1 expression in infected cells (Fig. 8). Moreover, in A72 uninfected cells, TCDD significantly increases the expression of CYP1A1 (data not shown).

These findings showed that CCoV infection in A72 cells upregulates protein expression of CYP1A1, which was further enhanced by TCDD.

## Discussion

Several findings suggest that exposure to different pollutants may influence the potential transmission of viruses, including CoVs (Domingo and Rovira 2020; Domingo et al. 2020b; Pozzer et al. 2020; Marquès and Domingo 2022; Bornstein et al. 2020; De Angelis et al. 2021; Barnett-Itzhaki and Levi 2021; Dales et al. 2021; Filippini et al. 2021). Indeed, the emergence of SARS-CoV-2, the virus associated with pandemic CoV disease 2019 (COVID-19), has also had a considerable impact on the study of the association between SARS-CoV-2 and contaminants in the environment. For instance, it has been recently demonstrated a positive correlation between chronic exposure to pollutants and SARS-CoV-2. Air pollutants, such as PM2.5, PM10, NO_2_, SO_2_, O_3_, and CO, are related to the incidence of COVID-19 cases, and the severity of symptoms in infected subjects (Domingo and Rovira 2020; Domingo et al. 2020b; Pozzer et al. 2020; Marquès and Domingo 2022). Furthermore, it has been detected that there is a noteworthy correlation between the level of environmental contaminants with the rate of mortality due to SARS-CoV-2, in some European and extra-European countries (Bornstein et al. 2020; Domingo and Rovira 2020; Domingo et al. 2020b; Marquès and Domingo 2022; De Angelis et al. 2021; Barnett-Itzhaki and Levi 2021; Dales et al. 2021; Filippini et al. 2021), highlighting the potential action of some pollutants in the incidence and severity of the disease (Domingo and Rovira 2020; Domingo et al. 2020b; Marquès and Domingo 2022). Moreover, in vivo and in vitro infection with MHV reveals an increase in virus titer in TCDD-exposed groups (Healey et al. 2024) as well as in genomic RNA (Grunewald et al. 2020), respectively. Thus, in this study, we evaluated the impact of a well-known environmental contaminant, like TCDD, on CCoV-II infection in a canine fibrosarcoma cell line (A72), suitable to explore AHR response to CCoV infection (Cerracchio et al. 2022, 2023, 2024), using an AHR specific agonist like TCDD. We first tested the effect of different doses of TCDD on cell viability. Herein, TCDD did not induce significant difference in a cancer cell line (A72) viability. Similar results have been previously described both in normal and cancer cells, like human embryonic fibroblast cell line (MRC-5) (Murayama et al. 2002), BMDMs (Grunewald et al. 2020), and human neuroblastoma cell line (SK-N-SH) (Luo et al. 2020) in which TCDD did not produce significant difference in cell viability/proliferation. Conversely, our previous results describe an increased cell viability of a bovine no cancer cell line, Madin Darby Bovine Kidney (MDBK) exposed to TCDD (Fiorito et al. 2008a, 2011). Moreover, Ahn et al., (2005) on non-tumorigenic immortalized human breast epithelial cells (M13SV1), reported an enhanced proliferation in TCDD-exposed cells. These results highlight the cell-type specificity response to TCDD exposure, with a variability in cell viability/proliferation assessment. Following CCoV infection in vitro, in the presence of TCDD, cells exhibited an enhancement of morphological cell death marks, due to the typical signs of cell death-induced during CCoV infection in A72 (De Martino et al. 2010; Ruggieri et al. 2007). Interestingly, these findings were accompanied by a dose-dependent increase in CCoV virus yield, with a strong growth in the expression of viral NP, a protein which has higher stability than spike protein in SARS-CoV-1 and SARS-CoV-2 (Tseng et al. 2021). Similarly, among CoVs, the TCDD-activation of AHR augments morbidity of mice infected with MHV, a prototypic CoV infection (Healey et al. 2024). In addition, in the presence of TCDD in mice infected with different viruses, unlike effects were reported. Specifically, in infection with influenza viruses, it has been described as an increased mortality of infected mice (House et al. 1990; Burleson et al. 1996; Bohn et al. 2005), as well as alteration in immune defenses against infection (Houser and Lawrence 2022). In addition, TCDD contributes to increase lethality in mice infected with herpes simplex II virus (Clark et al. 1983). Moreover, coxsackievirus B3 (CB3) infection modifies the uptake of TCDD into various tissues of the mouse (Funseth and Ilbäck 1994), and a redistribution of accumulated TCDD in organs was observed in mice infected with CB3 (Funseth et al. 2000). Furthermore, TCDD promotes virus replication in vitro of MHV (Grunewald et al. 2020), HIV-1 (Pokrovsky et al. 1991; Gollapudi et al. 1996), CMV (Murayama et al. 2002), and BoHV-1 (Fiorito et al. 2008a,b, 2010, 2017a). As previously reported (Cerracchio et al. 2022), during CCoV infection, we found an AHR activation, also described in other CoVs infection, such as MHV, MERS-CoV, HCoV-229E, HCoV-OC43, SARS-CoV-1, and SARS-CoV-2 (Giovannoni et al. 2021; Shi et al. 2023; Grunewald et al. 2020; Yousefi et al. 2023; Healey et al. 2024). To assess the impact of TCDD on AHR activation during CCoV infection in A72 cells, we have evaluated AHR and CYP1A1, an AHR target protein, whose expression levels in unexposed cells were upregulated during infection as reported in other CoVs infections, like HCoV-229E and SARS-CoV-1 (Giovannoni et al. 2021), SARS-CoV-2 (Giovannoni et al. 2021; Shi et al. 2023), as well as HCoV-OC43 (Yousefi et al. 2023). CYP1A1 during CCoV infection was enhanced by TCDD, according to the trends obtained following MHV infection in mice (Healey et al. 2024). Overall, our results could be a consequence of a modulation of AHR, observed also assessing a recognized effector downstream of AHR, like CYP1A1, that may be variably regulated by TCDD as well as by CCoV. The difference in expression levels of AHR signaling proteins might be due to a different level of saturation and/or degradation of receptor and its transcriptional targets (Pollenz 2002). Moreover, it has been established that the cellular response to viral infection is dependent on duration and timing of AHR signaling. For example, it has been shown that the timing of AHR activation significantly influences disease outcomes in viral infections. In a mouse model of herpes simplex virus type 1 (HSV-1) infection, pre-infection activation of AHR by TCDD resulted in increased mortality, whereas post-infection AHR activation ameliorated symptoms of herpetic encephalitis (Veiga-Parga et al. 2011). Similarly, the role of AHR modulation in shaping the humoral immune response has been reported in respiratory viral infections, highlighting the critical influence of timing on the host response to infection (Houser et al. 2024).

Here, we have presented the results obtained in cells that were contextually infected and exposed to TCDD. As above, TCDD, the most toxic and studied congener of PCDD/PCDFs family, was selected for our study. Further investigations are needed to clarify the mechanism by which an environmental contaminant, like TCDD, is involved in the regulation of CoVs’ infection.

## Conclusions

Our results prove that TCDD exposure could play a role as promoter of viral diseases, providing the findings of how environmental contaminants can impact host responses to CoVs’ infections and new insight on CCoV mechanism of action, underlining the regulation of virus replication by the AHR signaling.

## Data Availability

All data supporting the findings of this study are available within the paper.
